# Genome-Wide Identification of the PAL Gene Family in *Idesia polycarpa* and Transcriptomic Responses to *Botryosphaeria dothidea* Infection

**DOI:** 10.3390/ijms27177842

**Published:** 2026-09-02

**Authors:** Ming Liu, Qiuliang Chi, Han Zhao, Lisha Fang, Li Dai, Zhi Li, Xiaodong Geng, Juan Wang, Yongyu Ren, Yanmei Wang, Zhen Liu, Fangming Liu, Qifei Cai

**Affiliations:** 1 College of Forestry, Henan Agricultural University, Zhengzhou 450046, China; lmwyyi@163.com (M.L.); chiqiuliang08@163.com (Q.C.); daili@henau.edu.cn (L.D.); lizhi@henau.edu.cn (Z.L.); xiaodonggeng@163.com (X.G.); 15191419630@163.com (J.W.); yongyuren_1128@henau.edu.cn (Y.R.); wym3554710@163.com (Y.W.); liuzhen@henau.edu.cn (Z.L.); 2 Research Institute of Non-Timber Forestry, Chinese Academy of Forestry, Zhengzhou 450003, China; zhaohan@caf.ac.cn; 3 College of Forestry, Xinyang Agriculture and Forestry University, Xinyang 464000, China; lishafang0210@163.com; 4 National Forestry and Grassland Administration Key Laboratory for Central Plains Forest Resources Cultivation, Henan Agricultural University, Zhengzhou 450046, China; 5 Henan Province Engineering Technology Research Center for Idesia, Zhengzhou 450046, China

**Keywords:** *I. polycarpa*, PAL, *B. dothidea*, biotic stress, steady-state transcript abundance, woody plant pathology

## Abstract

*Idesia polycarpa* is a woody oil tree threatened by stem canker caused by *Botryosphaeria dothidea*, yet the organization and infection-responsive behavior of its phenylalanine ammonia-lyase (PAL) gene family remain poorly understood. Here, we identified five *IpPAL* genes and characterized their phylogenetic relationships, conserved features, duplication patterns, promoter cis-elements, and infection-associated expression profiles. Segmental and tandem duplication contributed to IpPAL family evolution, and all duplicated pairs showed Ka/Ks ratios below 1, consistent with purifying selection. RNA sequencing (RNA-seq) of contrasting Chengdu and Zhangjiajie provenances revealed distinct temporal responses. In Chengdu, *IpPAL2–IpPAL4* were significantly upregulated at 24 h after inoculation, whereas all five genes were upregulated at 96 h. In Zhangjiajie, all five *IpPAL* genes were significantly upregulated at 24 h, while *IpPAL2–IpPAL5* remained upregulated at 96 h. No *IpPAL* gene met the differential-expression criteria between provenances under mock conditions or at 24 h; at 96 h, *IpPAL1* and *IpPAL3* were lower and *IpPAL5* was higher in Zhangjiajie than in Chengdu. Scanning electron microscopy (SEM) provided complementary qualitative evidence of provenance-associated tissue responses. These findings demonstrate time- and gene-specific *IpPAL* responses to *B. dothidea* and identify candidate genes for further functional analysis.

## 1. Introduction

*Idesia polycarpa* is a woody oil tree distributed in Northeast Asia. Its fruits contain abundant oil rich in unsaturated fatty acids, supporting its development as an edible-oil and bioresource species [1,2,3]. Stem canker, however, is a major constraint on plantation health. In Henan Province, Fang et al. reported a disease incidence of 70.12% among 328 surveyed trees and identified *B. dothidea* as the causal pathogen [4]. Infection produces expanding bark lesions, cracking, internal tissue necrosis, and, in severe cases, branch dieback or tree mortality. Physiological work further indicates that the host response changes over the course of disease, with early antioxidant and osmotic responses followed by increasing oxidative and photosynthetic damage at later stages [5].

The phenylpropanoid pathway is one of the major metabolic pathways involved in plant responses to biotic stress. Its downstream metabolites, including lignin, flavonoids, and phenolic acids, contribute to disease resistance through cell wall reinforcement, antimicrobial activity, and regulation of defense-related processes [6,7]. Phenylalanine ammonia-lyase (PAL), generally encoded by a multigene family in plants, catalyzes the deamination of L-phenylalanine to trans-cinnamic acid and functions as the first rate-limiting enzyme in the phenylpropanoid pathway [8,9,10,11,12,13]. By playing a key role in directing metabolic flux through the phenylpropanoid pathway, PAL contributes to the biosynthesis of lignin, flavonoids, phenolic compounds, and other defense-related metabolites. The role of PAL in resistance to *B. dothidea* has been demonstrated in woody *Populus* species. In *Populus davidiana* × *P. alba* var. *pyramidalis*, the transcription factor PdpapWRKY11 activates the expression of *PdpapPAL* and *PdpapCAD,* thereby promoting lignin accumulation and enhancing resistance to *B. dothidea* [14]. In *Populus tomentosa*, PAL contributes to the supply of phenylalanine-derived precursors for salicylic acid (SA) biosynthesis. SA-associated defense signaling, including systemic acquired resistance (SAR), has been reported to restrict pathogen spread in woody plants, suggesting a potential role of PAL-mediated pathways in limiting *B. dothidea* infection within branches [15].

Beyond woody plants, PAL-dependent defense has been demonstrated across diverse pathosystems. *GhPAL9* contributes to *Verticillium* wilt resistance in cotton [16], *TaPAL* genes participate in lignin- and phenolic-associated resistance in wheat [17,18], *CaPAL3* and *CaPAL7* are linked to salicylic-acid-associated resistance in pepper [19], and PAL-dependent salicylic acid biosynthesis has been demonstrated in rice [20]. Pan-genome analysis in maize has also revealed temporal PAL-family responses to herbivory [21]. Collectively, these studies support PAL as a plausible component of inducible defense networks, but they also show that PAL regulation is strongly species-, genotype-, and stress-context dependent.

Our group previously identified *B. dothidea* strain SQ-40 from diseased *I. polycarpa* tissues [4] and, in a separate preliminary provenance screen that has not been published, observed contrasting canker phenotypes among geographic provenances. Zhangjiajie and Chengdu were selected as representative resistant and susceptible provenances, respectively, for the present molecular and microscopic analyses. Despite these phenotypic observations, the *PAL* gene complement, evolutionary history, and infection-associated steady-state RNA-abundance patterns have not been characterized at the genome-wide level in *I. polycarpa*. Moreover, because the two selected materials represent geographically distinct provenances, any basal transcriptional differences must be interpreted cautiously with respect to both disease phenotype and broader genetic background.

In this study, we systematically identified the *I. polycarpa* PAL family and examined physicochemical properties, gene structures, phylogenetic relationships, duplication events, and predicted cis-regulatory elements. We then integrated RNA sequencing (RNA-seq)abundance visualization with raw-count-based differential-expression analysis to characterize *IpPAL* responses in Zhangjiajie and Chengdu stem tissues at 0, 24, and 96 h after *B. dothidea* inoculation. *IpPAL* abundance was visualized using log_2_(FPKM + 1), whereas statistical differential-expression testing was performed on raw gene counts using DESeq2. Scanning electron microscopy (SEM) was used as complementary qualitative phenotyping to document host tissue microstructure and pathogen-associated surface morphology at corresponding stages. This design provides gene-level statistical support for the observed transcript-abundance patterns while recognizing that qRT-PCR, biochemical assays, and functional validation are still required to establish gene-specific mechanisms.

## 2. Results

### 2.1. Identification and Characterization of PAL Genes in Idesia polycarpa

Five IpPAL family members were identified in the I. polycarpa genome and named IpPAL1–IpPAL5 according to chromosomal position. IpPAL1 is located on Chr02, IpPAL2 on Chr15, IpPAL3 on Chr18, and IpPAL4 and IpPAL5 on Chr20 (Table S1). The predicted proteins are 711–716 aa long, with molecular weights of 77.51–77.83 kDa and theoretical pI values of 5.81–6.13. Their predicted instability indices are below 40 and GRAVY values are below 0. Bioinformatic localization prediction placed all five proteins in the cytoplasm, and no transmembrane domain or signal peptide was predicted. Detailed physicochemical properties and predicted subcellular localization information for the five IpPAL proteins are provided in Supplementary Table S2.

**Figure 1:**
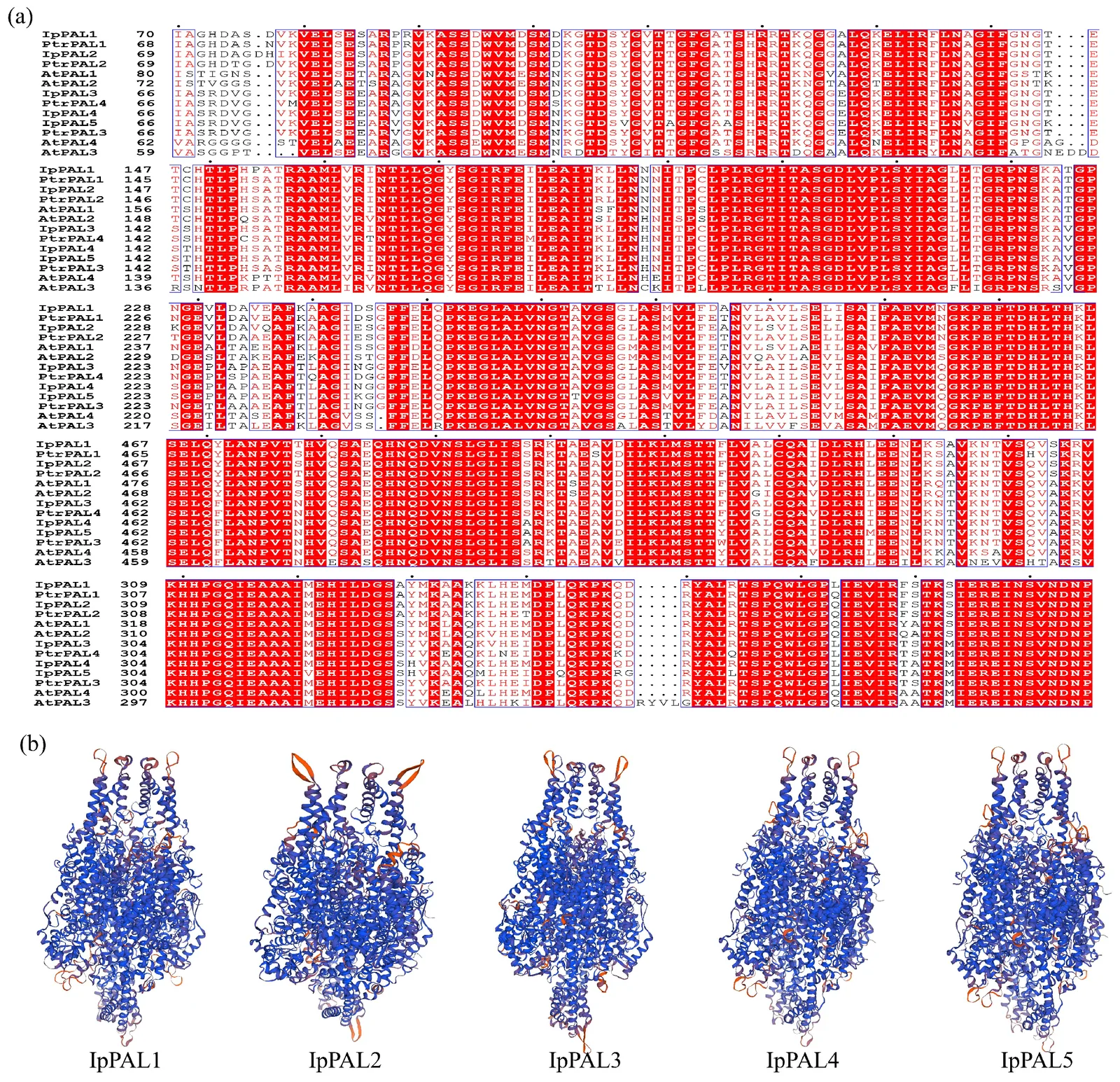
Bioinformatic sequence and structural comparison of IpPAL proteins. (**a**) Multiple sequence alignment of *IpPALs* with *AtPALs* and *PtrPALs*. Boxed regions indicate conserved regions associated with substrate recognition and catalysis, including the FL substrate-recognition motif, the MIO-forming Ala-Ser-Gly (ASG) motif within GTITASGDLVPLSYIA, and the conserved GLALVNG and HNQD motifs. (**b**) Predicted tertiary-structure models of the five IpPAL proteins generated using SWISS-MODEL. Structural models are presented as bioinformatic predictions and were not experimentally determined.

Multiple sequence alignment showed that all five IpPAL proteins contain the conserved ASG tripeptide, FL site, and characteristic PAL catalytic sequences (Figure 1a). NetPhos-based prediction indicated that putative phosphorylation sites occur predominantly at serine residues, with fewer threonine and tyrosine sites. SOPMA prediction indicated alpha-helices as the major secondary-structure component (54.43–55.84%), followed by random coils (36.39–39.80%) and extended strands (4.89–6.47%); no beta turns were predicted. SWISS-MODEL generated broadly similar predicted tertiary structures for all five proteins (Figure 1b). These outputs are computational predictions rather than experimentally verified protein properties; detailed phosphorylation and secondary-structure predictions are provided in Supplementary Table S3.

### 2.2. Phylogenetic Analysis of the PAL Family Genes

**Figure 2:**
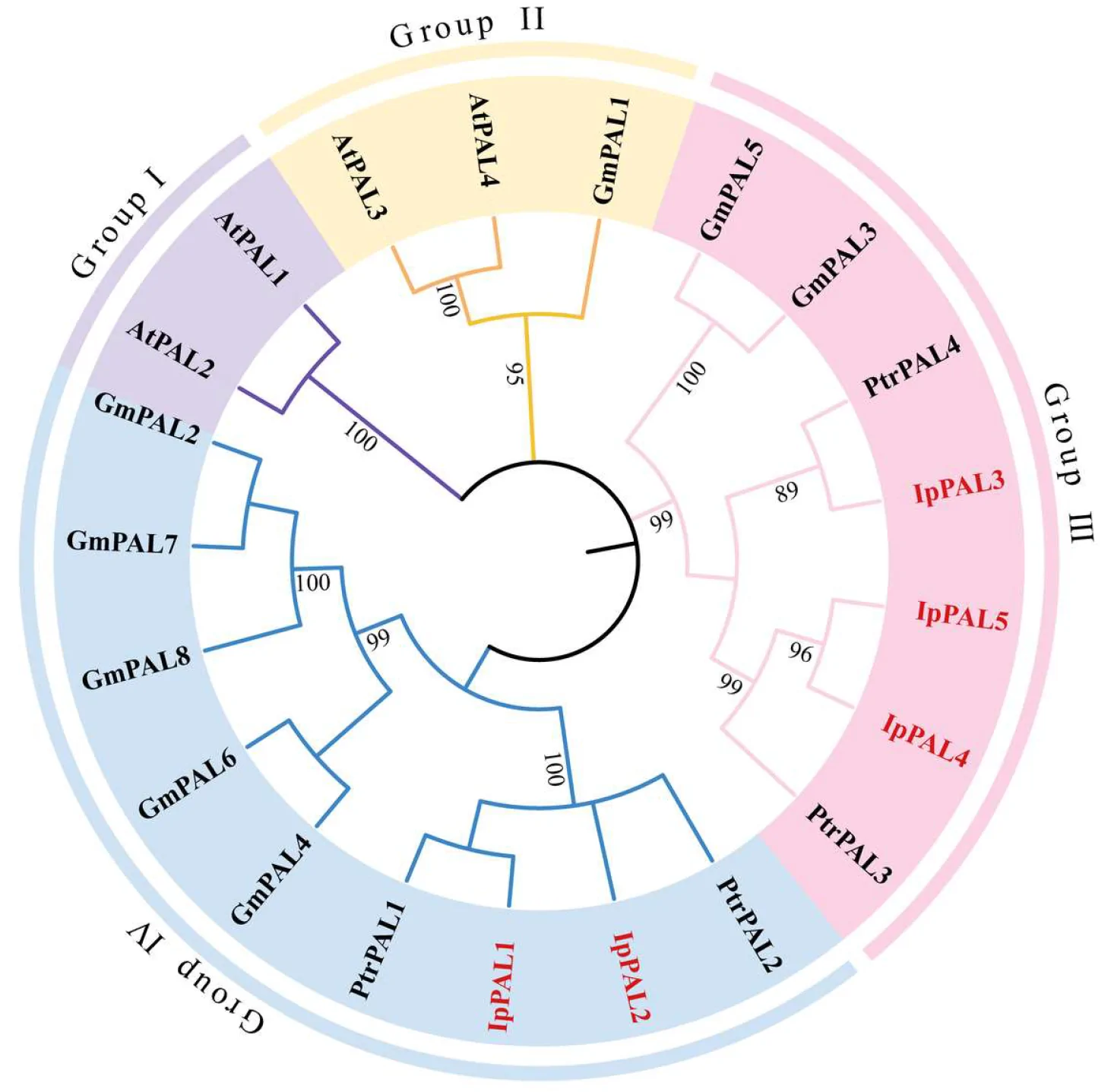
Neighbor-joining phylogenetic analysis of PAL proteins from *A. thaliana* (4 AtPALs), *I. polycarpa* (5 IpPALs, red labels), *G. max* (8 GmPALs), and *P. trichocarpa* (4 PtrPALs). The tree was generated with 1000 bootstrap replicates and divided into Groups I–IV. Numbers displayed at internal nodes are bootstrap percentages retained in the exported tree.

To elucidate the phylogenetic relationships among IpPALs, a neighbor-joining (NJ) phylogenetic tree was constructed using PAL protein sequences from *I. polycarpa* (five), *Arabidopsis thaliana* (four), *Populus trichocarpa* (four), and *Glycine max* (eight). A total of 21 PAL proteins were divided into Groups I–IV (Figure 2). Among them, IpPAL3, IpPAL4, and IpPAL5 were clustered in Group III, forming a monophyletic branch with high support together with PtrPAL2/4 from *P. trichocarpa* and GmPAL3/5 from *G. max*. IpPAL1 and IpPAL2 were distributed in Group IV, clustering with PtrPAL1/3 from *P. trichocarpa* and GmPAL2/4/6/7/8 from *G. max*. Group I consisted exclusively of AtPAL1/2 from *A. thaliana*, and Group II contained AtPAL3/4 from *A. thaliana* and GmPAL1 from *G. max*; neither group contained PAL sequences from *I. polycarpa* or *P. trichocarpa*.

### 2.3. Gene Structure and Conserved Motif Analysis of IpPAL Genes

**Figure 3:**
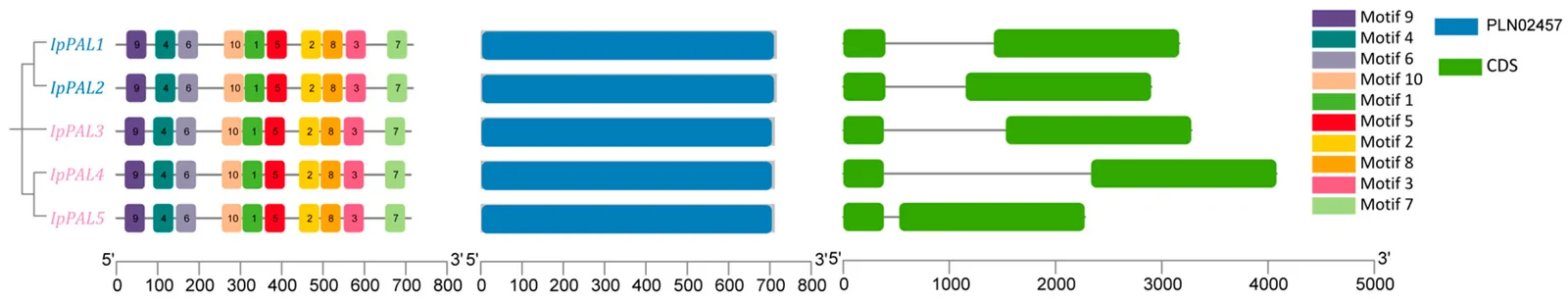
Conserved motif and gene structure analysis of *IpPAL* genes in *Idesia polycarpa*. Groups III and IV are marked with different colors on the left. From left to right: conserved motifs, domains, and CDS.

To analyze the structural characteristics of the *IpPAL genes* in *I. polycarpa*, their conserved motifs, protein domains, and intron distributions were examined (Figure 3). The results showed that the motif composition and arrangement patterns of the five IpPAL proteins were highly consistent, all containing Motif 1 to Motif 10, with no motif loss or gain. All IpPAL proteins contained a complete PLN02457 domain. Gene structure analysis revealed that the intron distribution patterns of IpPAL family members were relatively conserved, with variations occurring only in intron length.

### 2.4. Cis-Acting Element Analysis of IpPAL Gene Promoters

**Figure 4:**
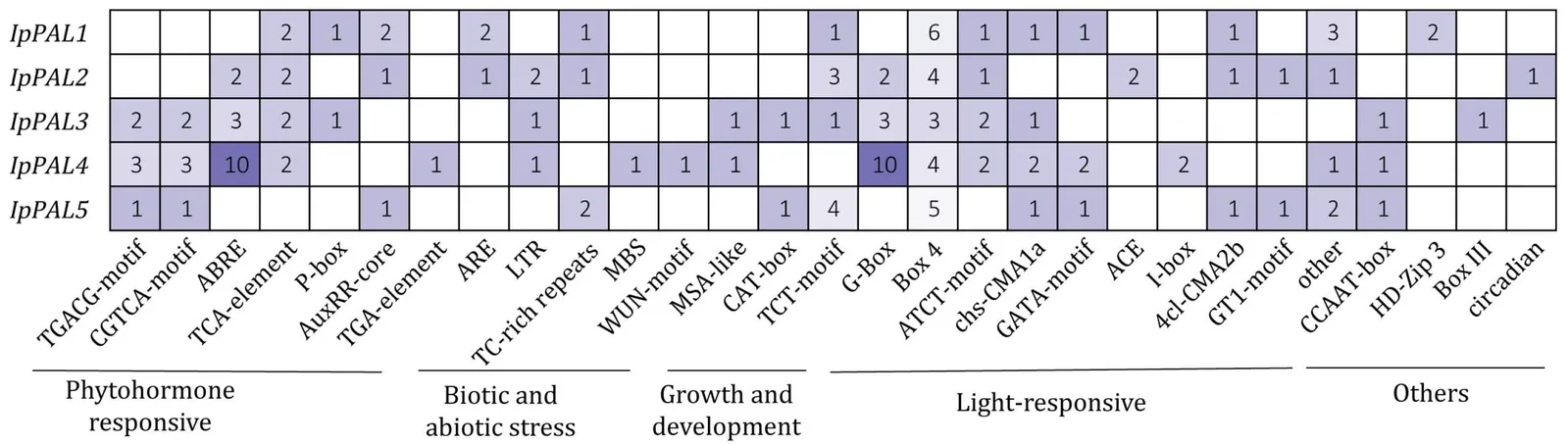
Predicted cis-acting elements in the *IpPAL* gene promoters. Elements are grouped as phytohormone-responsive, biotic/abiotic-stress-responsive, growth-and-development-related, light-responsive, and other elements. Cell shading represents the number of predicted elements at each promoter-element combination: darker purple indicates a larger count, whereas unshaded cells indicate zero.

To further investigate the potential functions of IpPALs, cis-element analysis was performed on their promoter regions (2000 bp upstream of the transcription start site). A total of 143 cis-elements were identified, which could be categorized into four major classes: light-responsive, phytohormone-responsive, biotic/abiotic stress-responsive, and growth and development-related elements (Table S4). Among them, light-responsive elements were the most abundant, with 78 elements accounting for 55% of the total, mainly including G-box (15) and Box 4 (22). A total of 42 phytohormone-responsive elements were identified, including TGACG-motif/CGTCA-motif (12), ABRE (15), TCA-element (8), P-box (2), and AuxRR-core/TGA-element (5). A total of 13 biotic/abiotic stress-responsive elements were identified, including ARE (3), LTR (4), TC-rich repeats (4), MBS (1), and WUN-motif (1). Three growth and development-related elements were identified, including MSA-like (2) and CAT-box (1). In addition, CCAAT-box (3), HD-Zip 3/Box III (3), and a circadian element (1) were also identified (Figure 4).

The number of elements in the promoters of IpPAL1 to IpPAL5 was 24, 25, 25, 47, and 22, respectively. All IpPAL gene promoters contained light-responsive elements (Box 4, G-box), hormone-responsive elements (ABRE, TCA-element, AuxRR-core), stress-responsive elements (ARE, LTR, TC-rich repeats), and growth and development-related elements (MSA-like, CCAAT-box). These elements represent potential regulatory sites associated with light signaling, hormone signaling, and stress responses. IpPAL4 had the highest number of elements and additionally contained MBS, WUN-motif, CAT-box, and a circadian element, suggesting that this gene may possess a more specific regulatory pattern in stress response and growth and development regulation.

### 2.5. Chromosomal Distribution and Collinearity Analysis of IpPAL Genes

**Figure 5:**
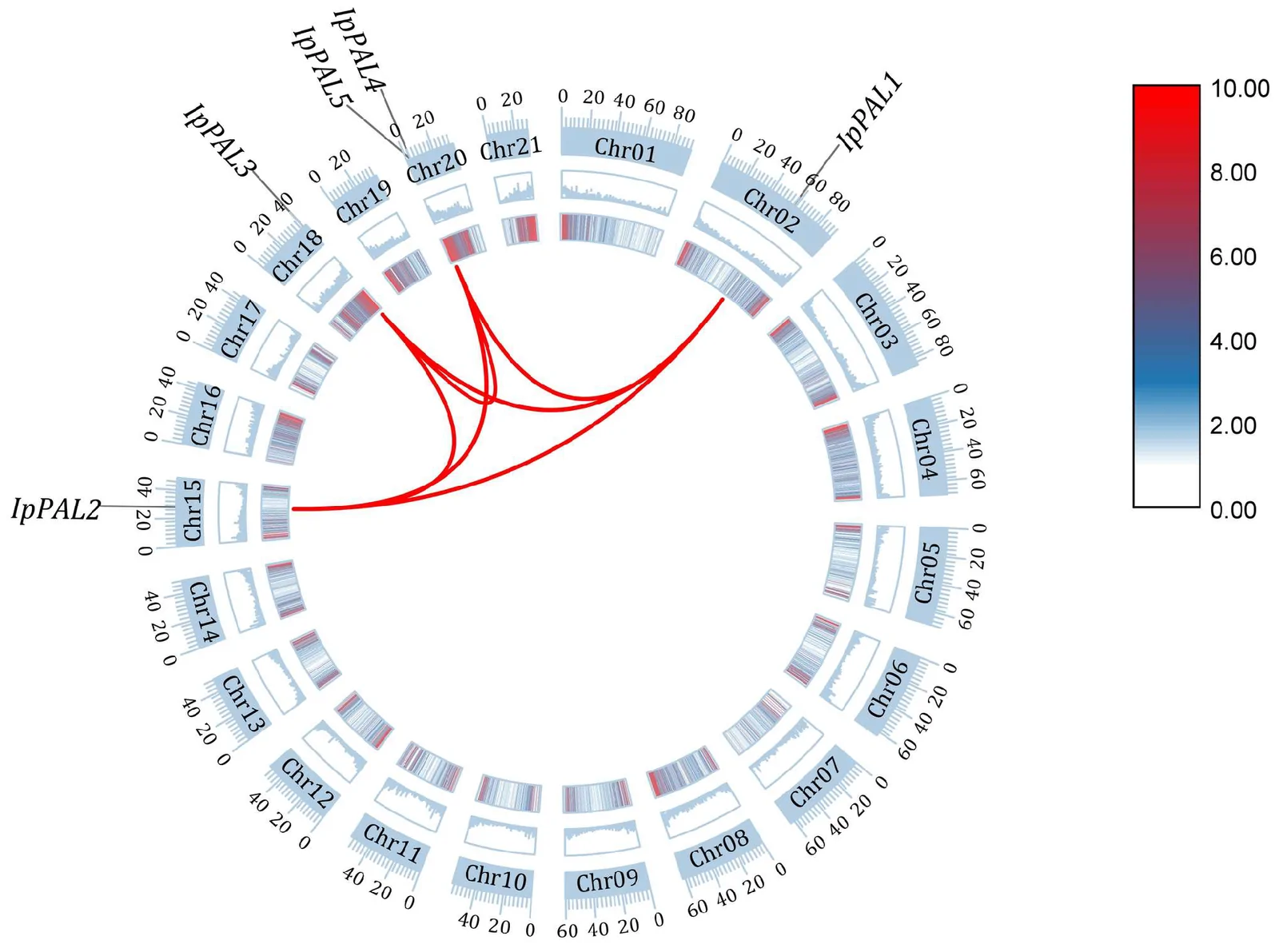
Chromosomal distribution and within-genome duplication relationships of *IpPAL* genes. Curved links connect identified *IpPAL* duplication pairs; chromosome labels, gene positions, gene-density tracks, and link colors are shown at the scale of the full *I. polycarpa* chromosome set.

Chromosomal localization analysis revealed that the five *IpPAL* genes were unevenly distributed on four chromosomes: *IpPAL1* was located on Chr02, *IpPAL2* on Chr15, *IpPAL3* on Chr18, and *IpPAL4* and *IpPAL5* were both located on Chr20 and formed a tandem duplication pair (Figure 5). Gene duplication is a critical first step in the functional expansion of multigene families. In the *IpPAL* genes, six segmentally duplicated gene pairs and one tandem duplication event were identified. This result indicates that segmental duplication and tandem duplication play key roles in the evolution of the *IpPAL* gene family. To assess the selective pressure acting on gene duplication events, the mutation rates between nonsynonymous (Ka) and synonymous (Ks) substitutions were compared. The Ka, Ks, and Ka/Ks ratios were calculated for the six gene pairs. All Ka/Ks ratios were below 1, indicating that the *IpPAL* genes have undergone purifying selection during evolution (Table S5).

**Figure 6:**
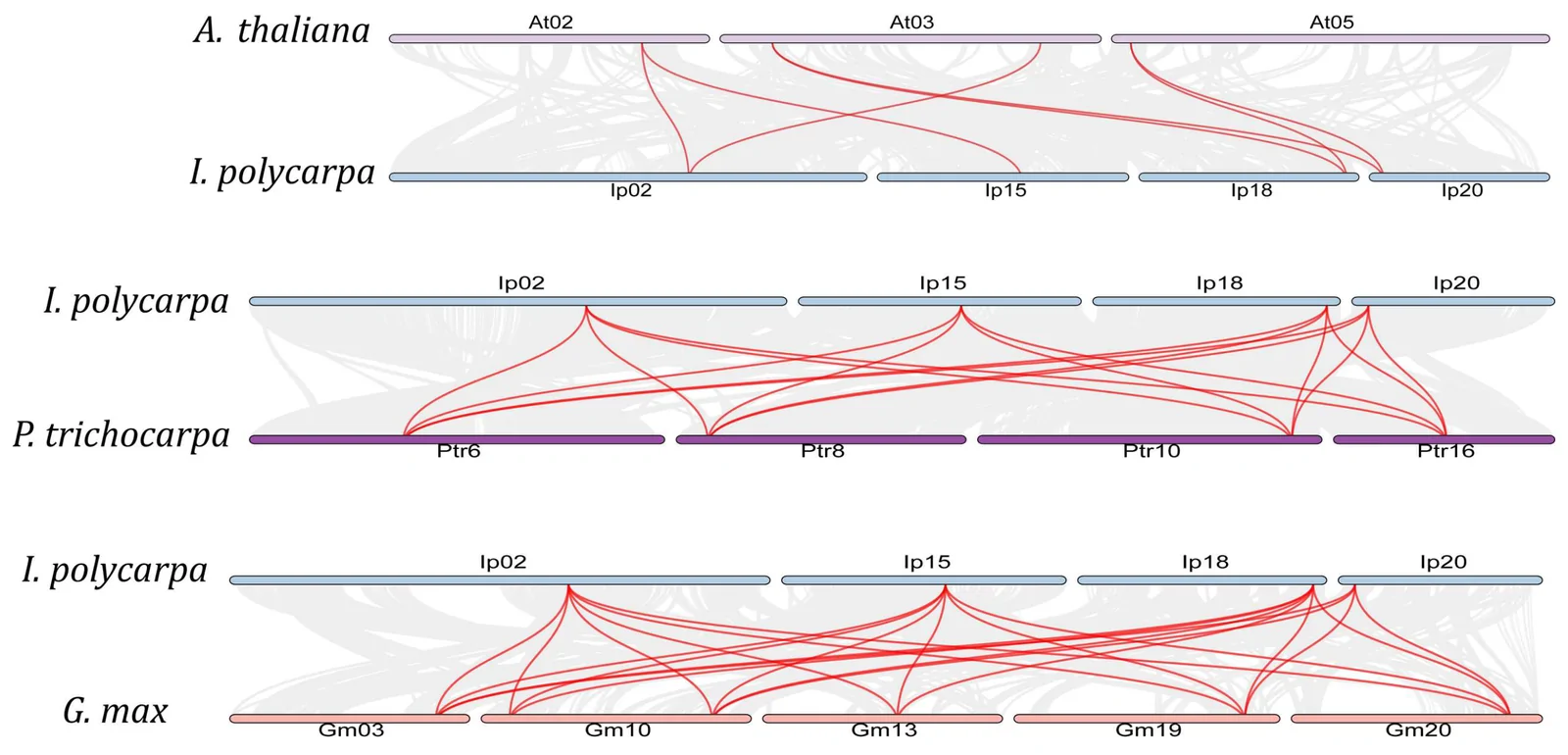
Graphical illustration of syntenic association of *I. polycarpa* with *A. thaliana*, *P. trichocarpa*, and *G. max*. Gray lines show all syntenic pairings, while red lines represent the syntenic association of *PAL* genes between *I. polycarpa* and the other three genomes.

To characterize conserved genomic neighborhoods without assuming simple one-to-one orthology, interspecific synteny analyses were performed between *I. polycarpa* and *A. thaliana*, *G. max*, and *P. trichocarpa*. 7, 22, and 16 PAL-associated syntenic links were detected with *A. thaliana*, *G. max*, and *P. trichocarpa*, respectively. *IpPAL1*, *IpPAL2*, *IpPAL3*, and IpPAL4 each participated in one or more cross-species syntenic relationships, and several *IpPAL* genes were linked to multiple homologous loci in the comparison genomes (Figure 6). These many-to-many syntenic relationships are consistent with lineage-specific duplication histories and should not be interpreted, by synteny alone, as evidence of strict one-to-one orthology.

### 2.6. IpPAL Transcript Abundance and Differential Expression Under B. dothidea Infection

The Zhangjiajie and Chengdu provenances were compared using log_2_ (FPKM + 1)-transformed abundance profiles for visualization, with the corresponding FPKM values provided in Supplementary Table S6, and raw-count-based DESeq2 analysis for statistical inference. Differentially expressed genes (DEGs) were defined using a fold-change threshold of ≥1.5 (or ≤1/1.5) and FDR < 0.05. At the transcriptome level, 12,260 and 13,303 DEGs were detected in CCK vs. C24 and CCK vs. C96, respectively, whereas 11,366 and 10,986 DEGs were identified in ZCK vs. Z24 and ZCK vs. Z96. Between the two provenances, the number of DEGs increased from 51 under mock-inoculated conditions to 5822 at 24 h and 9619 at 96 h, indicating increasing transcriptomic divergence during infection.

Gene-level analysis revealed distinct temporal responses of the *IpPAL* family. In Chengdu, *IpPAL2–IpPAL4* were significantly upregulated at 24 h, whereas all five *IpPAL* genes were significantly upregulated at 96 h. In Zhangjiajie, all five members were significantly upregulated at 24 h, while *IpPAL2–IpPAL5* remained significantly upregulated at 96 h. The strongest responses were observed for *IpPAL2* in Chengdu at 96 h (log_2_FC = 2.651, FDR = 7.74 × 10^−22^) and *IpPAL5* in Zhangjiajie at 96 h (log_2_FC = 1.988, FDR = 2.37 × 10^−19^).

**Figure 7:**
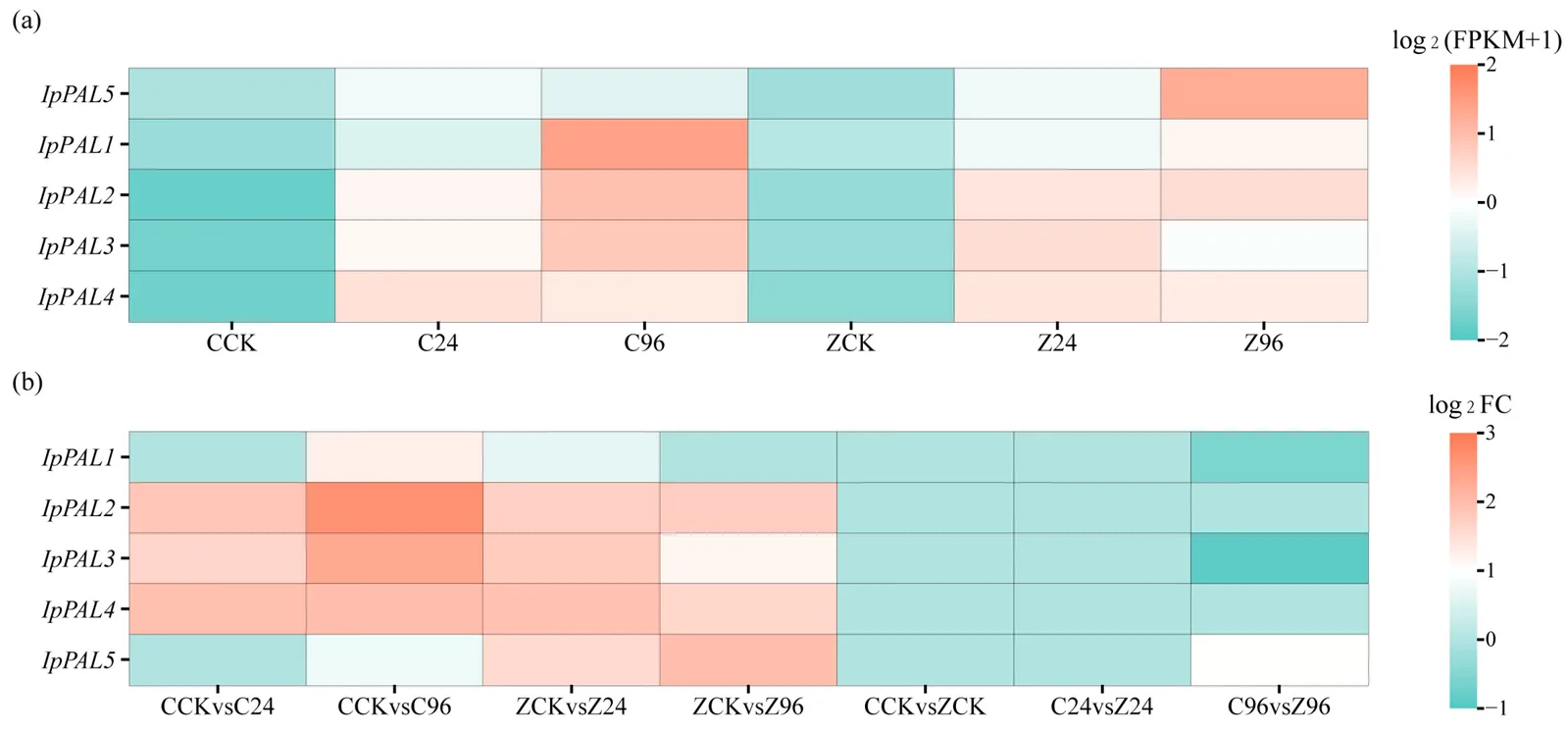
Steady-state transcript abundance and DESeq2-based differential expression of *IpPAL* genes in *Idesia polycarpa* following *Botryosphaeria dothidea* inoculation. (**a**) Heatmap of log_2_(FPKM + 1)-transformed steady-state transcript abundance for *IpPAL1–IpPAL5* in mixed stem tissues of the susceptible Chengdu and resistant Zhangjiajie provenances at 0, 24, and 96 h after inoculation. The corresponding FPKM values are provided in Supplementary Table S6. (**b**) Heatmap of DESeq2-derived gene-level log_2_ fold changes (log_2_FC) for *IpPAL1–IpPAL5* across seven pairwise comparisons. In each comparison (A vs. B), positive values indicate higher expression in B than in A, whereas negative values indicate lower expression in B than in A. FPKM values were used for normalized abundance visualization only, whereas differential expression was assessed independently from raw count data using DESeq2 with FDR correction. Detailed differential-expression statistics are listed in Supplementary Table S7. CCK and ZCK denote mock-inoculated controls of the Chengdu and Zhangjiajie provenances, respectively; C24/C96 and Z24/Z96 denote samples collected at 24 and 96 h post-inoculation, respectively. FPKM, fragments per kilobase of transcript per million mapped fragments; FDR, false discovery rate.

Direct comparisons between the two provenances showed that none of the five *IpPAL* genes met the DEG criteria under mock-inoculated conditions or at 24 h. At 96 h, however, *IpPAL1* and *IpPAL3* showed lower transcript abundance in Zhangjiajie than in Chengdu (log_2_FC = −0.587 and −0.883, respectively), whereas *IpPAL5* showed higher abundance (log_2_FC = 1.020). These results indicate a time-dependent and gene-specific *IpPAL* response, characterized by broader early activation in Zhangjiajie and clearer provenance-dependent divergence at 96 h. The corresponding log_2_ (FPKM + 1) abundance patterns and DESeq2-derived log_2_FC values are shown in Figure 7, with complete gene-level statistics provided in Supplementary Table S7.

### 2.7. Qualitative SEM Context for Host Structure and Fungal Colonization

**Figure 8:**
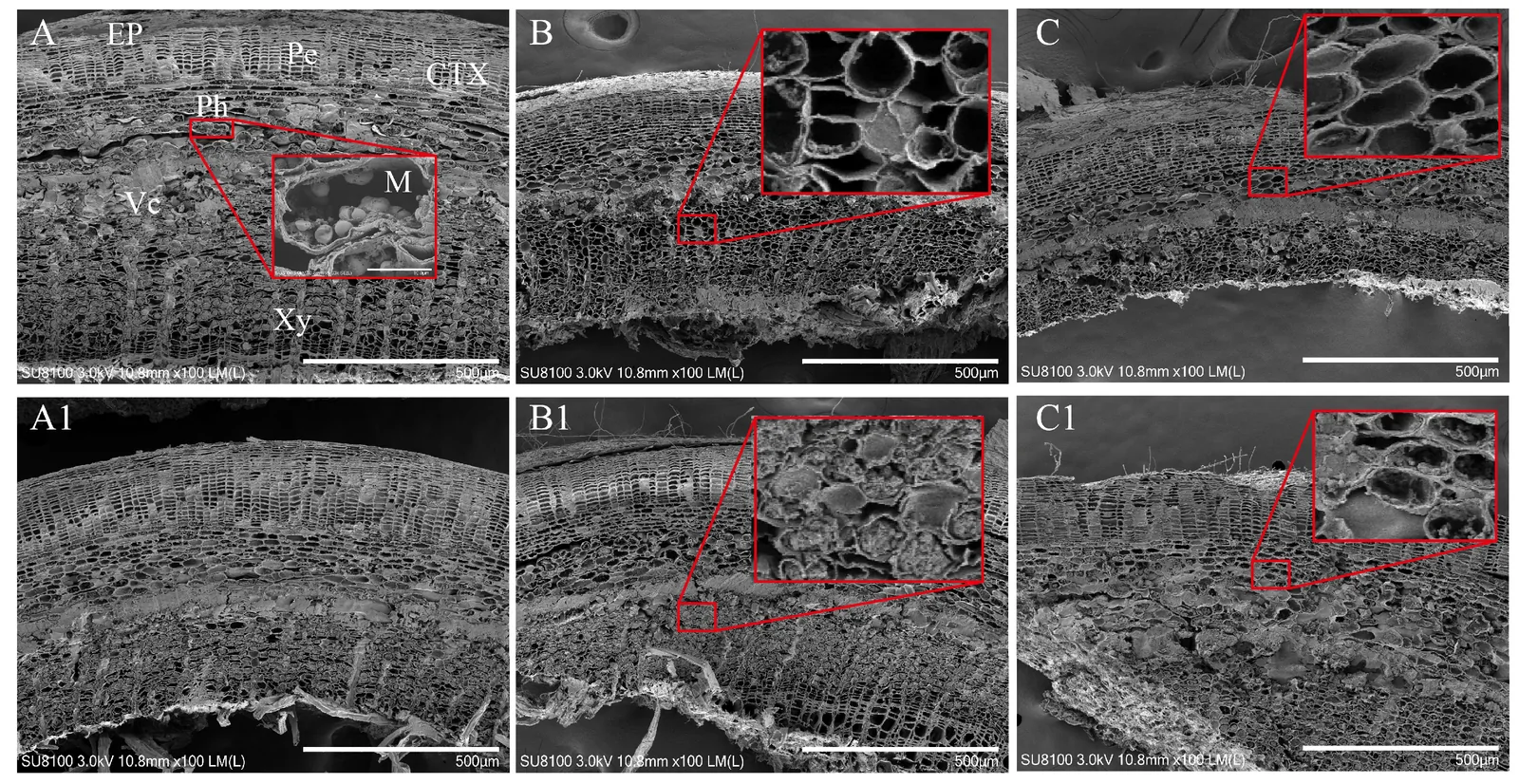
SEM characterization of stem tissue responses in susceptible Chengdu and resistant Zhangjiajie *Idesia polycarpa* provenances following *Botryosphaeria dothidea* inoculation. The upper row (**A**–**C**) shows the susceptible Chengdu provenance, whereas the lower row (**A1**–**C1**) shows the resistant Zhangjiajie provenance. (**A**,**A1**) represent mock-inoculated controls (CCK and ZCK, respectively); (**B**,**B1**) represent samples collected at 24 h post-inoculation (C24 and Z24); and (**C**,**C1**) represent samples collected at 96 h post-inoculation (C96 and Z96). Red boxes indicate selected regions that were digitally enlarged within the corresponding panels to facilitate visualization of local cellular and tissue structural features. These insets are digital enlargements of the original ×100 SEM images and do not represent independently acquired higher-magnification micrographs; therefore, no separate scale bars are assigned to the enlarged insets. The 500 μm scale bars shown at the bottom of the original SEM panels apply to the corresponding ×100 overview images. EP, epidermis; Pe, periderm; CTX, cortex; Ph, phloem; Vc, vascular cambium; Xy, xylem; M, granule-like inclusion.

SEM was included to provide tissue-level morphological context for the same contrasting provenances and infection stages; it was not used to establish a mechanistic link between *IpPAL* RNA abundance and tissue morphology. Under mock-inoculated conditions, the phloem–cambial and xylem regions of both provenances retained relatively intact organization, with numerous granule-like intracellular inclusions visible in the overview fields (Figure 8A,A1). These inclusions were not chemically identified, and their composition or metabolic function therefore cannot be inferred from SEM morphology alone.

After inoculation, qualitative differences were visible between the two provenances. At 24 h post-inoculation, fewer granule-like inclusions were visible in portions of the susceptible tissue field, whereas more remained visible in parts of the resistant xylem region (Figure 8B,B1). At 96 h, the susceptible field showed more extensive disruption of cellular organization, while portions of the resistant field retained comparatively clearer tissue architecture (Figure 8C,C1).

**Figure 9:**
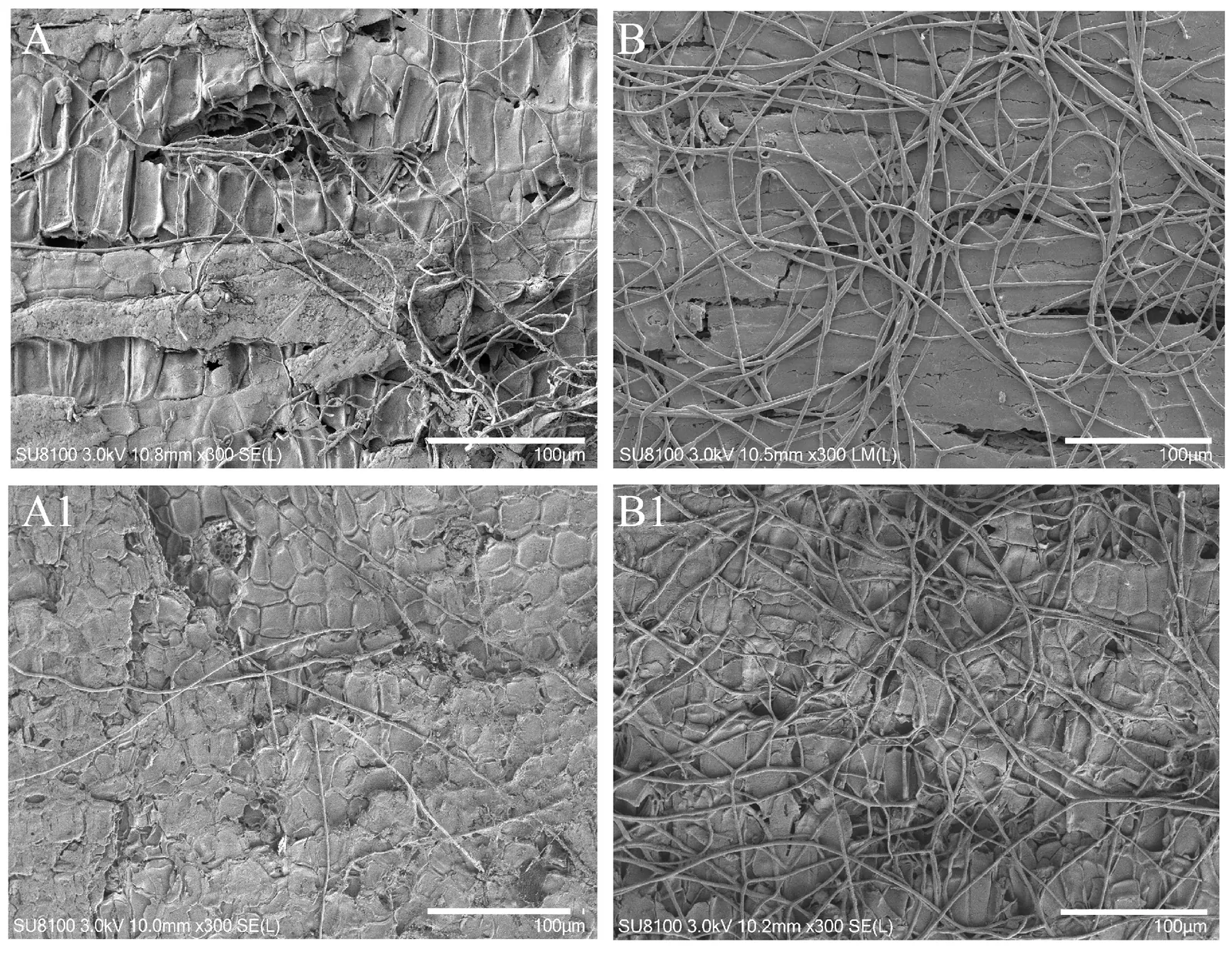
Representative ×300 SEM surface fields showing *B. dothidea*-associated filamentous structures at 24 and 96 h post-inoculation. (**A**) susceptible Chengdu at 24 h; (**A1**) resistant Zhangjiajie at 24 h; (**B**) susceptible Chengdu at 96 h; (**B1**) resistant Zhangjiajie at 96 h. The entire segmented horizontal bar immediately to the left of each “100 μm” label represents 100 μm. Instrument-generated scale bars and acquisition information are retained. Filamentous structures are described as hypha-like on morphological grounds; no quantitative hyphal-coverage or pathogen-biomass analysis was performed.

Complementary ×300 SEM surface fields showed filamentous structures morphologically consistent with fungal hyphae on infected tissues (Figure 9). At 24 h, more hypha-like structures were visible in the susceptible field than in the resistant field; by 96 h, abundant filamentous structures were present in both provenances, with more extensive surface disruption in the susceptible field. SEM alone cannot determine penetration route, hyphal coverage, or pathogen biomass; these panels are therefore interpreted as qualitative morphological observations rather than quantitative measures of disease progression.

## 3. Discussion

Plant *PAL* genes typically occur as small multigene families whose size varies among species. Four *PAL* genes have been reported in *A. thaliana* [22], eight in *G. max* [23], four in *P. trichocarpa* [24], and six each in *C. cathayensis* [25] and *C. nitidissima* [26]. The five *IpPAL* genes identified here therefore fall within the range reported for other dicots. The larger soybean family is consistent with the highly duplicated, palaeopolyploid soybean genome [27]. All five IpPAL proteins retain characteristic PAL sequence motifs, including the MIO-forming ASG motif and FL substrate-recognition site [28,29,30]. However, the phosphorylation sites, subcellular localization, secondary structures, and tertiary structures reported here are bioinformatic predictions. They support structural conservation at the hypothesis-generating level but cannot substitute for protein localization, post-translational modification, or structural validation experiments.

Phylogenetic and duplication analyses suggest that both segmental duplication and the tandem *IpPAL4*–*IpPAL5* pair contributed to the present family architecture, while Ka/Ks values below 1 are consistent with purifying selection [31,32]. The interspecific analysis further showed multiple PAL-associated syntenic links between *I. polycarpa* and the comparison genomes. Importantly, these links are many-to-many and are not equivalent to strict one-to-one orthology, particularly in genomes shaped by whole-genome and segmental duplication. The relatively high number of links with *G. max* is therefore compatible with soybean genome duplication rather than evidence for an unusually large set of simple orthologs. Promoter analyses also predicted hormone-, stress-, and light-responsive cis-elements [33], but the regulatory activity of these elements must be tested experimentally.

The count-based DESeq2 analysis substantially refines interpretation of the *IpPAL* heatmap. Within Chengdu, three *IpPAL* genes (*IpPAL2*–*IpPAL4*) were significantly upregulated at 24 h, whereas all five members were upregulated at 96 h. In Zhangjiajie, all five *IpPAL* genes were significantly upregulated at 24 h, and four members (*IpPAL2*–*IpPAL5*) remained upregulated at 96 h. This pattern is consistent with a broader early activation of the *IpPAL* family in Zhangjiajie and a broader late response in Chengdu. Importantly, however, direct between-provenance comparisons identified no *IpPAL* genes meeting the DEG criteria under mock conditions or at 24 h. Therefore, the numerical differences visible in the FPKM heatmap at these stages should not be interpreted as statistically significant between-provenance expression differences. Provenance-dependent divergence became evident at 96 h, when *IpPAL1* and *IpPAL3* were lower in Zhangjiajie than in Chengdu, whereas *IpPAL5* was higher. These results argue against a simple model in which resistance is associated with constitutively higher or uniformly stronger PAL-family expression; instead, *IpPAL* responses appear temporally and gene-specifically differentiated. Temporal heterogeneity in defense-associated transcription has likewise been reported in maize herbivory and *Castanea–Phytophthora* interactions [34,35]. Recent pear studies further emphasize that *B. dothidea* resistance is controlled by broader regulatory networks: *PcMYB44* can enhance lignification and defense-gene expression [36], while the *MSTRG.32189*–*PcmiR399b*–*PcUBC24* module links phosphate status to reactive-oxygen-species-associated disease resistance [37]. These observations support evaluating PAL as one component of a multilayer defense network rather than as an isolated causal determinant. In addition, Zhangjiajie and Chengdu are geographically distinct provenances; their transcriptional differences may reflect resistance-associated states, broader population-genetic background, or both.

The SEM observations were intentionally retained as complementary pathology context. The observed disruption of vascular-associated tissues is broadly consistent with structural damage reported for woody canker pathosystems [38,39]. However, the granule-like inclusions in Figure 8 were not chemically identified, and no starch, soluble-carbon, phenylpropanoid, or metabolite measurements were performed at the SEM sampling points. We therefore do not infer carbon balance, storage status, or defense-metabolic capacity from these structures. Likewise, the apparent differences in hypha-like structures in Figure 9 are qualitative and should not be interpreted as measured differences in pathogen biomass. Their value in the present study is to document the tissue environment in which the RNA-abundance profiles were sampled.

Several limitations define the next experimental steps. First, although raw-count DESeq2 analysis provides inferential support for RNA-seq differences, independent qRT-PCR validation of *IpPAL1*–*IpPAL5* remains important for confirming gene-specific expression patterns. Second, only two geographically distinct provenances were analyzed; a larger provenance panel or segregating population will be required to separate disease-resistance effects from broader genetic-background differences. Third, PAL function is regulated beyond RNA abundance: trans-cinnamic acid can feed back on PAL activity [40], Kelch repeat F-box proteins control PAL turnover [41,42], and phosphorylation or subcellular localization can alter PAL function [43]. Measurements of PAL protein abundance and enzyme activity, lignin/phenolic metabolites, pathogen biomass, and functional perturbation of priority *IpPAL* genes will therefore be necessary to establish the mechanism. Within these limits, the present work provides a genome-level catalog together with statistically analyzed transcriptomic responses and a focused set of IpPAL candidates for downstream validation.

## 4. Materials and Methods

### 4.1. Identification of IpPAL Family Members in Idesia polycarpa

The chromosome-level *I. polycarpa* reference genome described by Zuo et al. [44] and its annotation files were obtained from the National Genomics Data Center (NGDC; https://ngdc.cncb.ac.cn/gwh/, accessed on 29 August 2026). PAL protein sequences of *A. thaliana* were retrieved from The Arabidopsis Information Resource (TAIR; https://www.arabidopsis.org/, accessed on 29 August 2026), and the PAL-domain HMM profile (PF00221) was obtained from Pfam. *A. thaliana* PAL proteins were first used as BLASTP (version 2.17.0) queries against the *I. polycarpa* protein set (E-value < 1 × 10^−5^). HMMER 3.0 was then used to screen for additional candidate PAL proteins. Redundant candidates were removed, and the remaining sequences were checked against NCBI CDD for the complete PAL conserved domain before final family assignment.

Physicochemical properties were predicted using ExPASy ProtParam (https://web.expasy.org/protparam/, accessed on 29 August 2026); subcellular localization using WoLF PSORT (https://wolfpsort.hgc.jp/, accessed on 29 August 2026); phosphorylation sites using NetPhos 3.1; secondary structure using SOPMA (https://npsa.lyon.inserm.fr/cgi-bin/npsa_automat.pl?page=/NPSA/npsa_sopma.html, accessed on 29 August 2026); and tertiary structure using SWISS-MODEL. Multiple-sequence alignment was performed with ClustalW 2.1 (https://www.genome.jp/tools-bin/clustalw, accessed on 29 August 2026) and visualized with ESPript 3 (https://espript.ibcp.fr/ESPript/ESPript/, accessed on 29 August 2026). Throughout this manuscript, outputs from these tools are explicitly described as predictions rather than experimentally verified features.

### 4.2. Phylogenetic, Gene Duplication, and Synteny Analysis

PAL protein sequences of *P. trichocarpa* and *G. max* were retrieved from NCBI. The PAL amino acid sequences were aligned with ClustalW, and a neighbor-joining tree was constructed in MEGA 12 with 1000 bootstrap replicates. The exported tree was visualized in iTOL. Whole-genome sequences and annotations of *A. thaliana*, *G. max*, and *P. trichocarpa* were obtained from Ensembl Plants. Intraspecific and interspecific synteny analyses were performed in TBtools v2.472 using the integrated MCScanX workflow, and Ka/Ks values were calculated with the TBtools simple Ka/Ks calculator.

### 4.3. Gene Structure, Conserved Motif, and Conserved Domain Analysis

Gene structure analysis was performed using TBtools. Conserved motifs were predicted using the MEME tool (https://meme-suite.org/meme/, accessed on 29 August 2026) with the number of motifs set to 10. Conserved domains were identified using the NCBI CDD tool (https://www.ncbi.nlm.nih.gov/Structure/bwrpsb/bwrpsb.cgi, accessed on 29 August 2026). All analysis results were integrated and visualized using TBtools.

### 4.4. Cis-Acting Element Analysis

To analyze cis-acting elements, 2000 bp promoter sequences upstream of the transcription start site of each *IpPAL* gene were extracted using TBtools software. Cis-acting elements in the promoter regions were predicted using the PlantCARE online tool (http://bioinformatics.psb.ugent.be/webtools/plantcare/html/, accessed on 29 August 2026).

### 4.5. Pathogen Material and Selection of Contrasting Provenances

*Botryosphaeria dothidea* strain SQ-40 was isolated from naturally infected stem tissues of *I. polycarpa*, and its identity and pathogenicity were established in our previously published study [4]. The ITS, TEF1, and TUB sequences of this isolate are available in GenBank under accession numbers OQ748086, OQ815803, and OQ815811, respectively. For routine maintenance, SQ-40 was cultured on potato dextrose agar (PDA) until the colony completely covered the Petri dish, after which the culture plates were stored at 4 °C. Before inoculation, the stored culture was revived at 25 °C for approximately 48 h, transferred onto fresh PDA medium, and incubated in darkness at 25 °C for 5 d. Five-millimeter-diameter mycelial plugs were excised from the actively growing colony margins and used for inoculation.

Two geographically distinct *I. polycarpa* provenances maintained in the Henan Agricultural University germplasm collection were used in this study: Zhangjiajie and Chengdu. These two provenances had shown contrasting responses to *B. dothidea* infection in a preliminary resistance assessment conducted before the RNA-seq and SEM experiments. The preliminary assessment was used only to establish the contrasting disease phenotypes of the two plant materials and to support their selection for subsequent comparative analyses.

Five healthy trees were represented for each provenance. Six uniform, disease-free, 2-year-old branches were selected across these trees for each experimental replicate, and three independent replicates were performed. Branches were trimmed to approximately 25 cm in length, washed with running water, surface-sterilized with 75% ethanol, rinsed three times with sterile water, and sealed at both ends with paraffin wax. A single wound approximately 5 mm in diameter was made on each branch segment using a sterile punch. A 5 mm mycelial plug was placed mycelium-side down onto each wound, whereas sterile PDA plugs were used for mock inoculation. The inoculation sites were covered with sterile water-moistened cotton, and the branches were maintained at 25 °C under humid conditions and sprayed with sterile water three times daily.

Longitudinal lesion length was measured 15 d after inoculation. Lesions were assigned to seven ordinal classes according to lesion length: <0.5, 0.5–1.0, 1.0–1.5, 1.5–2.0, 2.0–2.5, 2.5–3.0, and >3.0 cm, corresponding to grade values of 0–6. The disease index (DI) was calculated as Σ(number of inoculation sites at each grade × grade value)/(total number of inoculation sites × 6) × 100. The preliminary assessment showed contrasting disease responses between the two provenances. Zhangjiajie exhibited a relatively resistant phenotype, with a mean lesion length of approximately 0.8 ± 0.4 cm and a disease index (DI) of approximately 16, whereas Chengdu exhibited a highly susceptible phenotype, with a mean lesion length of approximately 3.0 ± 1.2 cm and a DI of approximately 81. These unpublished preliminary data were used only to establish the contrasting disease phenotypes and to select plant materials for the subsequent RNA-seq and SEM analyses; they were not treated as outcome data of the present transcriptomic or microscopic experiments.

### 4.6. RNA-Seq Library Construction, Sequencing, and Differential-Expression Analysis

For the transcriptome experiment, the same branch-wounding and inoculation procedure was applied to the Zhangjiajie and Chengdu provenances. Six experimental groups were analyzed: resistant mock control (ZCK), resistant samples collected at 24 and 96 h post-inoculation (Z24 and Z96), susceptible mock control (CCK), and susceptible samples collected at 24 and 96 h post-inoculation (C24 and C96), with three biological replicates per group. At each sampling time, approximately 1 mm^3^ of mixed stem tissue spanning the bark/phloem, cambial region, and outer xylem surrounding the inoculation site was collected, immediately frozen in liquid nitrogen, and stored at −80 °C until RNA extraction.

Total RNA extraction, library construction, and sequencing were performed by Biomarker Technologies Co., Ltd. (Beijing, China). RNA purity, concentration, and integrity were evaluated before library preparation. Eukaryotic mRNA was enriched from total RNA using oligo(dT)-conjugated magnetic beads and randomly fragmented. First- and second-strand cDNA synthesis was subsequently performed, followed by end repair, A-tailing, sequencing-adapter ligation, fragment-size selection using AMPure XP beads, and PCR enrichment to generate poly(A)-selected mRNA libraries. Qualified libraries were sequenced on an Illumina NovaSeq 6000 platform (Yeasen Biotechnology (Shanghai) Co., Ltd., Shanghai, China) using 150-bp paired-end sequencing (PE150).

Raw sequencing reads were subjected to quality filtering before downstream analysis. Reads containing adapter sequences were removed, together with reads in which ambiguous nucleotides (N) accounted for more than 10% of the total read length and reads in which more than 50% of bases had Phred quality scores of Q ≤ 10. After filtering, a total of 116.71 Gb of clean data was obtained from the 18 libraries, with at least 5.78 Gb of clean data generated per sample and Q30 percentages of ≥89.51%.

Clean paired-end reads were aligned to the *I. polycarpa* reference genome (Idesia_polycarpa.customer_v1.genome.fa) using HISAT2 [45]. The overall mapping rates among the 18 libraries ranged from 73.37% to 90.98%, with a mean mapping rate of approximately 83.17%. Mapped reads were assembled and quantified using StringTie [46]. Gene and transcript abundance were normalized as fragments per kilobase of transcript per million mapped fragments (FPKM), and log_2_(FPKM + 1) values were used for heatmap visualization. For inferential differential-expression analysis, gene-level raw count data were analyzed using DESeq2 [47] because all comparison groups contained three biological replicates. Comparisons followed the A_vs._B convention, with A treated as the reference/control group and B as the comparison/treatment group; positive log_2_FC therefore indicates higher abundance in group B relative to group A. Genes with a fold change ≥1.5 for upregulation or ≤1/1.5 for downregulation and an FDR-adjusted *p* value < 0.05 were classified as differentially expressed. DESeq2-derived log_2_FC and FDR values were used for statistical inference, whereas FPKM values were used only for normalized abundance visualization and not for significance testing. The filtered DEG output contained genes meeting both the fold-change and FDR criteria; therefore, gene-comparison combinations absent from these files are reported only as not meeting the predefined DEG criteria.

### 4.7. Scanning Electron Microscopy

For the present SEM experiment, approximately 1 mm^3^ host tissue blocks were excised directly from the margin of the inoculation wound at the mock-control, 24 h, and 96 h sampling points. This sampling position was chosen anatomically; a separate distance from the visible lesion or hyphal-colonization front was not recorded. Samples were gently rinsed with PBS, fixed in electron-microscopy fixative for 2 h at room temperature, stored at 4 °C, washed with 0.1 M phosphate buffer (PB; pH 7.4), post-fixed in 1% OsO4 in 0.1 M PB for 1–2 h in the dark, washed, dehydrated through graded ethanol and isoamyl acetate, critical-point dried, and sputter-coated with gold for 30 s. Samples were examined with an SU8100 SEM at 3.0 kV. Transverse-section overview fields were acquired at ×100 with instrument-generated 500 μm scale bars; complementary surface fields at 24 and 96 h were acquired at ×300 with instrument-generated 100 μm scale bars. The complete segmented bar adjacent to each scale label denotes the stated distance.

## 5. Conclusions

Five *IpPAL* genes were identified in the *I. polycarpa* genome and showed conserved PAL-family sequence features, while duplication and synteny analyses defined their evolutionary context. Raw-count DESeq2 analysis demonstrated clear time-dependent *IpPAL* responses to *B. dothidea*. In Zhangjiajie, all five *IpPAL* genes were significantly upregulated at 24 h and *IpPAL2*–*IpPAL5* remained upregulated at 96 h; in Chengdu, *IpPAL2*–*IpPAL4* were upregulated at 24 h and all five members were upregulated at 96 h. Direct comparisons between provenances identified no *IpPAL* DEGs under mock conditions or at 24 h, whereas at 96 h *IpPAL1* and *IpPAL3* were lower and *IpPAL5* was higher in Zhangjiajie relative to Chengdu. These results support temporally and gene-specifically differentiated PAL-family responses rather than a uniformly stronger response in the resistant provenance. SEM documented qualitative differences in tissue organization and pathogen-associated morphology and serves as complementary pathological context rather than mechanistic evidence for PAL function. Together, these data identify prioritized *IpPAL* candidates for qRT-PCR validation, PAL enzyme/protein assays, phenylpropanoid measurements, pathogen-biomass quantification, and functional genetic testing.

## Data Availability

The original contributions presented in this study are included in the article/Supplementary Material. Further inquiries can be directed to the corresponding authors.

## Supplementary Materials

The following supporting information can be downloaded at https://www.mdpi.com/article/10.3390/ijms27177842/s1.
