# Supplementary material for: Genome-Wide Identification of the PAL Gene Family in Idesia polycarpa and Transcriptomic Responses to Botryosphaeria dothidea Infection
Source: Int J Mol Sci. 2026 Sep 2;27(17):7842. doi: 10.3390/ijms27177842 (PMC13565970; doi:10.3390/ijms27177842)
Supplement: Supplementary file 1 [file ijms-27-07842-s001.zip › ijms-4525899-supplementary.pdf]

Supplementary Document

Genome-Wide Identification of the PAL Gene Family in *Idesia polycarpa* and Transcriptomic Responses to *Botryosphaeria dothidea* Infection

Ming Liu 1,†, Qiuliang Chi 1,†, Han Zhao 2, Lisha Fang 3, Li Dai 1,4,5, Zhi Li 1,4,5, Xiaodong Geng 1,4,5, Juan Wang 1,4,5, Yongyu Ren 1,4,5, Yanmei Wang 1,4,5, Zhen Liu 1,4,5, Fangming Liu 1,4,5,* and Qifei Cai 1,4,5,*

1 College of Forestry, Henan Agricultural University, Zhengzhou 450046, China

2 Research Institute of Non-Timber Forestry, Chinese Academy of Forestry, Zhengzhou 450003, China

3 College of Forestry, Xinyang Agriculture and Forestry University, Xinyang 464000, China

4 National Forestry and Grassland Administration Key Laboratory for Central Plains Forest Resources Cultivation, Henan Agricultural University, Zhengzhou 450046, China

5 Henan Province Engineering Technology Research Center for Idesia, Zhengzhou 450046, China

* Correspondence: fangmingliu-0930@henau.edu.cn (F.L.); cai_qifei@henau.edu.cn (Q.C.)

† These authors contributed equally to this work.

Table S1. Protein sequences of the IpPALs identified in this study.

Gene name	Gene ID	Sequence type	Sequences
IpPAL1	Idesia.02 G01482.t1	Protein	METITKNGYQNGSSESLCTSNLHDPLNWGVAAEAMKGSHLDEVKRMVAEYRKPVVNLGG<br>QTLTIAQVASIAGHDASDVKVELSESARPRVKASSDWVMDSMDKGTDSYGVTTGFGATSH<br>RRTKQGGALQKELIRFLNAGIFGNGTETCHTLPHPATRAAMLVRINTLLQGYSGIRFEILEAI<br>TKLLNNNITPCLPLRGTITASGDLVPLSYIAGLLTGRPNSKATGPNGEVLDAVEAFKAAGIDS<br>GFFELQPKEGLALVNGTAVGSGLASMVLFDANVLAVLSELISAIFAEVMNGKPEFTDHLTH<br>KLKHHPGQIEAAAIMEHILDGSAYMKAAKKLHEMDPLQKPKQDRYALRTSPQWLGPLIEVI<br>RFSTKSIEREINSVNDNPLIDVSRNKALHGGNFQGTPIGVSMDNVRLAIASIGKLLFAQFSEL<br>VNDFYNNGLPSNLTASRNPSLDYGFKGAEIAMASYCSELQYLANPVTTHVQSAEQHNQDV<br>NSLGLISSRKTAEAVDILKLMSTTFLVALCQAIDLRHLEENLKSAVKNTVSQVSKRVLTTGA<br>NGELHPSRFCEKELLQVVDREYVFAYADDPCSATYPLMQKLRQVLVDHALANGENEKNTS<br>TSVFQKIGAFEEELKALLPKEVESARAACDSGNSAIENKIKECRSYPLYKFVREELGTGLLTG<br>EKVRSPGEEFDKVFTAMCQGRIIDPMLECLGEWNGAPLPIC*
IpPAL2	Idesia.15 G00946.t1	Protein	MESITKNGYENGSSESLCTNQHDPLNWGVAAEAMKGSHLDEVKRMVAEYRKPVVKLGGE<br>SLTIAQVASIAGHDAGDHIKVELSESARPGVKASSDWVMDSMDKGTDSYGVTTGFGATSH<br>RRTKQGGALQRELIRFLNAGIFGNGTETCHTLPHSATRAAMLVRINTLLQGYSGIRFEILEAI<br>TKLLNNNITPCLPLRGTITASGDLVPLSYIAGLLTGRPNSKATGPKGEVLDAVQAFKAAGIES<br>GFFELQPKEGLALVNGTAVGSGLASMVLFEANVLSVLSELLSAIFAEVMNGKPEFTDHLTH<br>KLKHHPGQIEAAAIMEHILDGSAYMKAAKKLHEMDPLQKPKQDRYALRTSPQWLGPQIEVI<br>RFSTKSIEREINSVNDNPLIDVSRNKALHGGNFQGTPIGVSMDNVRLAIASIGKLLFAQFSEL<br>VNDFYNNGLPSNLTASRNPSLDYGFKGAEIAMASYCSELQYLANPVTSHVQSAEQHNQDV<br>NSLGLISSRKTAEAVDILKLMSTTFLVALCQAIDLRHLEENLKSAVKNTVSQVSKRVLTTGA<br>NGELHPSRFCEKELLKVVDIEYVFAYADDPCSATYPLMQKLRQVLVDHALANGENEKNLS<br>TSVFQKIEAFEEELKALLPKEVESARAAYDSGNSAIDNKIKECRSYPLYKFVREELGTGLLTG<br>ENILSPGEEFDKVFTAMCQGKIIDPMLECLGEWNGSPLPIC*
IpPAL3	Idesia.18 G02014.t1	Protein	MEFCQDSCIGNGSLGFNTNDPLNWGMMAESLKGSHLDEVKRMIEEYRKPVVKLGGETLTI<br>AQVTAIASRDVGVKVELSEEARAGVKASSDWVMDSMDKGTDSYGVTTGFGATSHRRTKQ<br>GGELQKELIRFLNAGIFGNGTESSHTLPHSATRAAMLVRINTLLQGYSGIRFEILEAITKLLNH<br>NITPCLPLRGTITASGDLVPLSYIAGLLTGRPNSKAVGPNGEPLAPAEAFTLAGINGGFFELQP<br>KEGLALVNGTAVGSGLASMVLFEVNVLAILSEVISAIFAEVMQGKPEFTDHLTHKLKHHPG<br>QIEAAAIMEHILDGSSYVKAAQKVHEIDPLQKPKQDRYALRTSPQWLGPLIEVIRTSTKMIER<br>EINSVNDNPLIDVSRNKALHGGNFQGTPIGVSMDNTRLAIASIGKLMFAQFSELVNDFYNNG<br>LPSNLTGGRNPSLDYGFKGAEIAMASYCSELQFLANPVTNHVQSAEQHNQDVNSLGLISSR<br>KTAEAVDILKLMSTTFLVALCQAIDLRHIEENLKNTVKNTVSQVAKRVLTMGFNGELHPSR<br>FCEKDLLKVVDREHVFAYIDDPCSATYPLMQKLRQVLVDHALMNGEKEKISTTSIFQKIESF<br>EEELKTLLPKEVESARLEVENGNPAIPNRIKECRSYPLYRFVREELGTSLLTGEKVRSPGEEF<br>DKVFTAICAGKLIDPLLECLKEWNGAPLPIC*
IpPAL4	Idesia.20 G00276.t1	Protein	MEFCQGSCNGNGSLGFNANDPLNWGMLAESLKGSHLDEVKRMIDEYRKPVVKLGGENLTI<br>GQVTAIASRDVGVKVELSEEARVGVKASSDWVMDSMNKGTDSYGVTTGFGATSHRRTKQ<br>GGELQKELIRFLNAGIFGNGTESTHTLPHSATRAAMLVRINTLLQGYSGIRFEILEAITKLLNH<br>NITPCLPLRGTITASGDLVPLSYIAGLLTGRPNSKAVGPSGEPLAPAEAFTLAGINGGFFELQP<br>KEGLALVNGTAVGSGLASMVLFETNVLAILSEVLSAIFAEVMQGKPEFTDHLTHKLKHHPG

			QIEAAAIMEHILDGSSHVKAAQKLHEMDPLQKPKQDRYALRTSPQWLGPLIEVIRTATKMIE<br>REINSVNDNPLIDVSRNKALHGGNFQGTPIGVSMDNTRLAIASIGKLMFAQLSELVNDYYNN<br>GLPSNLTGGRNPSLDYGFKGAEIAMASYCSELQFLANPVTNHVQSAEQHNQDVNSLGLISA<br>RKTAEAVDILKLMSTTYLVALCQAIDLRHIEENLKNTVKNTVSQVAKRVLTMGFNGELHPS<br>RFCEKDLLKVVDREHVFAYIDDPCSATYPLMQKLRQALVDHALRNGEKEKNSSTSIFQKIG<br>AFEEELKTLLPKEVEGARLELENGNPAIPNRIKECRSYPLYKFVREELGTSLLTGENVRSPGE<br>EFDKVFTAICAGKLIDPMLECLKEWNGAPLPLC*
IpPAL5	Idesia.20<br>G00277.t1	Protein	MEFCQDSCNSNGSLGFNANDPLNWCVVAESLKGSHLDEVKRMIDEYRKPVVKLGGENLTI<br>GQVTAIASRDVGVKVELSEEARVGVKASSDWVMDSMNKGTDSVGVTAGFGAASHRKTKQ<br>GGELQKELIRFLNVGIFGNGKESTHTLPHSATRAAMLVRINTLLQGYSGIRFEILEAITKLLN<br>HNITPCLPLRGTITASGDLVPLSYIAGLLTGRPNSKAVGPSGEPLAPAEAFTLAGIKGGFFELQ<br>PKEGLALVNGTTVGSGLASVVLFEANVLAILSEVLSAIFAEVMQGKPEFTDHLTHKLKHHP<br>GQIEAAAIVEHILDGSSHVKAAQMLHEIDPQQKPKRGRYALLTSPQWLGPLIEVIRTATKMI<br>EREINSVNDNPLIDVSRNKALYGGNFQGTPIGVSMDNTRLAIASIGKLMFAQFSELVNDYYN<br>NGLPSNLTGGRNPSLDYGFKGAEVAMASYCSELQFLANPVTNHVQSAEQHNQDVNSLGLIS<br>ARKTAEAVDILKLMSTTYLVALCQAIDLRHMEENLRNTVKNTVSQVAKRVLKMCFDAELH<br>PSGFCEKDLLKVVDREHVFAYIDDPCSATYPLMQKLRQALVDHALTNDEKEKNSSTSIFQKI<br>GAFEEELKTLLPKEVESARLELENGNPAIPNRIKECRSYPLYKFVREELGTSLLTGENVRSPG<br>EEFDKVFTAICAGKLIDPMLECLKEWNGAPLPIC*

Table S2. Predicted physicochemical properties and subcellular localization of IpPAL proteins.

Gene	Amino acids	Molecular weight (kDa)	pI	Instability index	Aliphatic index	GRAVY	Predicted localization
IpPAL1	716	77.78	5.96	33.27	90.08	-0.177	Cytoplasm
IpPAL2	716	77.83	5.81	34.77	90.63	-0.182	Cytoplasm
IpPAL3	711	77.8	5.9	34.28	92.73	-0.158	Cytoplasm
IpPAL4	711	77.53	6.13	31.72	91.93	-0.194	Cytoplasm
IpPAL5	711	77.51	5.97	31.25	93.42	-0.148	Cytoplasm

Table S3. Predicted phosphorylation-site counts and secondary-structure composition of IpPAL proteins.

Gene	Serine sites	Threonine sites	Tyrosine sites	α-helix (%)	Random coil (%)	Extended strand (%)	Beta turn (%)
IpPAL1	34	17	10	55.73	36.39	4.89	0
IpPAL2	39	15	10	55.17	39.53	5.31	0
IpPAL3	34	19	6	55.84	37.97	6.19	0
IpPAL4	31	17	7	54.43	39.8	5.77	0
IpPAL5	30	16	5	54.99	38.54	6.47	0

Table S4 Prediction of *cis*-regulatory elements in promoter regions of IpPAL*s*

Gene Name	Element Name	Sequence	Function Description
IpPAL4	TGA-element	AACGAC	auxin-responsive element
IpPAL3	MSA-like	(T/C)C(T/C)AACGG(T/C)(T/C)A	cis-acting element involved in cell cycle regulation
IpPAL4	MSA-like	TCCAACGGT	cis-acting element involved in cell cycle regulation
IpPAL1	TC-rich repeats	GTTTTCTTAC	cis-acting element involved in defense and stress responsiveness
IpPAL2	TC-rich repeats	GTTTTCTTAC	cis-acting element involved in defense and stress responsiveness
IpPAL5	TC-rich repeats	GTTTTCTTAC	cis-acting element involved in defense and stress responsiveness
IpPAL5	TC-rich repeats	ATTCTCTAAC	cis-acting element involved in defense and stress responsiveness
IpPAL2	ACE	CTAACGTATT	cis-acting element involved in light responsiveness
IpPAL2	ACE	CTAACGTATT	cis-acting element involved in light responsiveness
IpPAL2	LTR	CCGAAA	cis-acting element involved in low-temperature responsiveness
IpPAL2	LTR	CCGAAA	cis-acting element involved in low-temperature responsiveness
IpPAL3	LTR	CCGAAA	cis-acting element involved in low-temperature responsiveness
IpPAL4	LTR	CCGAAA	cis-acting element involved in low-temperature responsiveness
IpPAL1	TCA-element	CCATCTTTTT	cis-acting element involved in salicylic acid responsiveness
IpPAL1	TCA-element	CCATCTTTTT	cis-acting element involved in salicylic acid responsiveness
IpPAL2	TCA-element	TCAGAAGAGG	cis-acting element involved in salicylic acid responsiveness
IpPAL2	TCA-element	CCATCTTTTT	cis-acting element involved in salicylic acid responsiveness
IpPAL3	TCA-element	TCAGAAGAGG	cis-acting element involved in salicylic acid responsiveness
IpPAL3	TCA-element	CCATCTTTTT	cis-acting element involved in salicylic acid responsiveness
IpPAL4	TCA-element	CCATCTTTTT	cis-acting element involved in salicylic acid responsiveness
IpPAL4	TCA-element	CCATCTTTTT	cis-acting element involved in salicylic acid responsiveness
IpPAL2	ABRE	ACGTG	cis-acting element involved in the abscisic acid responsiveness
IpPAL2	ABRE	ACGTG	cis-acting element involved in the abscisic acid responsiveness
IpPAL3	ABRE	CACGTG	cis-acting element involved in the abscisic acid responsiveness
IpPAL3	ABRE	ACGTG	cis-acting element involved in the abscisic acid responsiveness
IpPAL3	ABRE	ACGTG	cis-acting element involved in the abscisic acid responsiveness
IpPAL4	ABRE	ACGTG	cis-acting element involved in the abscisic acid responsiveness
IpPAL4	ABRE	GACACGTACGT	cis-acting element involved in the abscisic acid responsiveness
IpPAL4	ABRE	ACGTG	cis-acting element involved in the abscisic acid responsiveness
IpPAL4	ABRE	ACGTG	cis-acting element involved in the abscisic acid responsiveness
IpPAL4	ABRE	ACGTG	cis-acting element involved in the abscisic acid responsiveness
IpPAL4	ABRE	GACACGTGGC	cis-acting element involved in the abscisic acid responsiveness
IpPAL4	ABRE	CACGTG	cis-acting element involved in the abscisic acid responsiveness
IpPAL4	ABRE	ACGTG	cis-acting element involved in the abscisic acid responsiveness
IpPAL4	ABRE	ACGTG	cis-acting element involved in the abscisic acid responsiveness
IpPAL4	ABRE	ACGTG	cis-acting element involved in the abscisic acid responsiveness
IpPAL1	ARE	AAACCA	cis-acting regulatory element essential for the anaerobic induction

IpPAL1	ARE	AAACCA	cis-acting regulatory element essential for the anaerobic induction
IpPAL2	ARE	AAACCA	cis-acting regulatory element essential for the anaerobic induction
IpPAL1	AuxRR-core	GGTCCAT	cis-acting regulatory element involved in auxin responsiveness
IpPAL1	AuxRR-core	GGTCCAT	cis-acting regulatory element involved in auxin responsiveness
IpPAL2	AuxRR-core	GGTCCAT	cis-acting regulatory element involved in auxin responsiveness
IpPAL5	AuxRR-core	GGTCCAT	cis-acting regulatory element involved in auxin responsiveness
IpPAL2	circadian	CAAAGATATC	cis-acting regulatory element involved in circadian control
IpPAL2	G-box	CACGTC	cis-acting regulatory element involved in light responsiveness
IpPAL2	G-Box	CACGTT	cis-acting regulatory element involved in light responsiveness
IpPAL3	G-Box	CACGTG	cis-acting regulatory element involved in light responsiveness
IpPAL3	G-box	CACGTG	cis-acting regulatory element involved in light responsiveness
IpPAL3	G-box	TACGTG	cis-acting regulatory element involved in light responsiveness
IpPAL4	G-box	TACGTG	cis-acting regulatory element involved in light responsiveness
IpPAL4	G-box	CACGTC	cis-acting regulatory element involved in light responsiveness
IpPAL4	G-box	CACGTC	cis-acting regulatory element involved in light responsiveness
IpPAL4	G-box	tgACACGTGGCA	cis-acting regulatory element involved in light responsiveness
IpPAL4	G-box	GCCACGTGGA	cis-acting regulatory element involved in light responsiveness
IpPAL4	G-box	CACGTG	cis-acting regulatory element involved in light responsiveness
IpPAL4	G-box	TACGTG	cis-acting regulatory element involved in light responsiveness
IpPAL4	G-box	TACGTG	cis-acting regulatory element involved in light responsiveness
IpPAL4	G-Box	CACGTT	cis-acting regulatory element involved in light responsiveness
IpPAL4	G-Box	CACGTG	cis-acting regulatory element involved in light responsiveness
IpPAL3	CGTCA-motif	CGTCA	cis-acting regulatory element involved in the MeJA-responsiveness
IpPAL3	CGTCA-motif	CGTCA	cis-acting regulatory element involved in the MeJA-responsiveness
IpPAL4	CGTCA-motif	CGTCA	cis-acting regulatory element involved in the MeJA-responsiveness
IpPAL4	CGTCA-motif	CGTCA	cis-acting regulatory element involved in the MeJA-responsiveness
IpPAL4	CGTCA-motif	CGTCA	cis-acting regulatory element involved in the MeJA-responsiveness
IpPAL5	CGTCA-motif	CGTCA	cis-acting regulatory element involved in the MeJA-responsiveness
IpPAL3	TGACG-motif	TGACG	cis-acting regulatory element involved in the MeJA-responsiveness
IpPAL3	TGACG-motif	TGACG	cis-acting regulatory element involved in the MeJA-responsiveness
IpPAL4	TGACG-motif	TGACG	cis-acting regulatory element involved in the MeJA-responsiveness
IpPAL4	TGACG-motif	TGACG	cis-acting regulatory element involved in the MeJA-responsiveness

IpPAL4	TGACG-motif	TGACG	cis-acting regulatory element involved in the MeJA-responsiveness
IpPAL5	TGACG-motif	TGACG	cis-acting regulatory element involved in the MeJA-responsiveness
IpPAL5	O2-site	GATGATGTGG	cis-acting regulatory element involved in zein metabolism regulation
IpPAL5	O2-site	GATGATGTGG	cis-acting regulatory element involved in zein metabolism regulation
IpPAL3	CAT-box	GCCACT	cis-acting regulatory element related to meristem expression
IpPAL5	CAT-box	GCCACT	cis-acting regulatory element related to meristem expression
IpPAL5	AT-rich sequence	TAAAATACT	element for maximal elicitor-mediated activation (2copies)
IpPAL1	HD-Zip 1	CAAT(A/T)ATTG	element involved in differentiation of the palisade mesophyll cells
IpPAL1	P-box	CCTTTTG	gibberellin-responsive element
IpPAL3	P-box	CCTTTTG	gibberellin-responsive element
IpPAL1	4cl-CMA2b	TCTCACCAACCACA	light responsive element
IpPAL2	4cl-CMA2b	TCTCACCAACCCCA	light responsive element
IpPAL5	4cl-CMA2b	TCTCACCAACCACA	light responsive element
IpPAL2	GT1-motif	GGTTAA	light responsive element
IpPAL5	GT1-motif	GGTTAAT	light responsive element
IpPAL4	MBS	CAACTG	MYB binding site involved in drought-inducibility
IpPAL1	MRE	AACCTAA	MYB binding site involved in light responsiveness
IpPAL3	CCAAT-box	CAACGG	MYBHv1 binding site
IpPAL4	CCAAT-box	CAACGG	MYBHv1 binding site
IpPAL5	CCAAT-box	CAACGG	MYBHv1 binding site
IpPAL1	ATCT-motif	AATCTAATCC	part of a conserved DNA module involved in light responsiveness
IpPAL2	ATCT-motif	AATCTAATCC	part of a conserved DNA module involved in light responsiveness
IpPAL3	ATCT-motif	AATCTAATCC	part of a conserved DNA module involved in light responsiveness
IpPAL3	ATCT-motif	AATCTAATCC	part of a conserved DNA module involved in light responsiveness
IpPAL4	ATCT-motif	AATCTAATCC	part of a conserved DNA module involved in light responsiveness
IpPAL4	ATCT-motif	AATCTAATCC	part of a conserved DNA module involved in light responsiveness
IpPAL1	Box 4	ATTAAT	part of a conserved DNA module involved in light responsiveness
IpPAL1	Box 4	ATTAAT	part of a conserved DNA module involved in light responsiveness
IpPAL1	Box 4	ATTAAT	part of a conserved DNA module involved in light responsiveness

IpPAL1	Box 4	ATTAAT	part of a conserved DNA module involved in light responsiveness
IpPAL1	Box 4	ATTAAT	part of a conserved DNA module involved in light responsiveness
IpPAL1	Box 4	ATTAAT	part of a conserved DNA module involved in light responsiveness
IpPAL2	Box 4	ATTAAT	part of a conserved DNA module involved in light responsiveness
IpPAL2	Box 4	ATTAAT	part of a conserved DNA module involved in light responsiveness
IpPAL2	Box 4	ATTAAT	part of a conserved DNA module involved in light responsiveness
IpPAL2	Box 4	ATTAAT	part of a conserved DNA module involved in light responsiveness
IpPAL3	Box 4	ATTAAT	part of a conserved DNA module involved in light responsiveness
IpPAL3	Box 4	ATTAAT	part of a conserved DNA module involved in light responsiveness
IpPAL3	Box 4	ATTAAT	part of a conserved DNA module involved in light responsiveness
IpPAL4	Box 4	ATTAAT	part of a conserved DNA module involved in light responsiveness
IpPAL4	Box 4	ATTAAT	part of a conserved DNA module involved in light responsiveness
IpPAL4	Box 4	ATTAAT	part of a conserved DNA module involved in light responsiveness
IpPAL4	Box 4	ATTAAT	part of a conserved DNA module involved in light responsiveness
IpPAL5	Box 4	ATTAAT	part of a conserved DNA module involved in light responsiveness
IpPAL5	Box 4	ATTAAT	part of a conserved DNA module involved in light responsiveness
IpPAL5	Box 4	ATTAAT	part of a conserved DNA module involved in light responsiveness
IpPAL5	Box 4	ATTAAT	part of a conserved DNA module involved in light responsiveness
IpPAL5	Box 4	ATTAAT	part of a conserved DNA module involved in light responsiveness
IpPAL1	chs-CMA1a	TTACTTAA	part of a light responsive element
IpPAL3	chs-CMA1a	TTACTTAA	part of a light responsive element
IpPAL4	chs-CMA1a	TTACTTAA	part of a light responsive element
IpPAL4	chs-CMA1a	TTACTTAA	part of a light responsive element
IpPAL5	chs-CMA1a	TTACTTAA	part of a light responsive element
IpPAL1	GA-motif	ATAGATAA	part of a light responsive element

IpPAL1	Gap-box	CAAATGAA(A/G)A	part of a light responsive element
IpPAL1	GATA-motif	AAGATAAGATT	part of a light responsive element
IpPAL4	GATA-motif	AAGATAAGATT	part of a light responsive element
IpPAL4	GATA-motif	AAGGATAAGG	part of a light responsive element
IpPAL5	GATA-motif	GATAGGG	part of a light responsive element
IpPAL4	I-box	gGATAAGGTG	part of a light responsive element
IpPAL4	I-box	ccttatcct	part of a light responsive element
IpPAL5	LAMP-element	CTTTATCA	part of a light responsive element
IpPAL2	L-box	attctcaCCTACCA	part of a light responsive element
IpPAL5	L-box	ATCCCACCTAC	part of a light responsive element
IpPAL1	TCCC-motif	TCTCCCT	part of a light responsive element
IpPAL1	TCT-motif	TCTTAC	part of a light responsive element
IpPAL2	TCT-motif	TCTTAC	part of a light responsive element
IpPAL2	TCT-motif	TCTTAC	part of a light responsive element
IpPAL2	TCT-motif	TCTTAC	part of a light responsive element
IpPAL3	TCT-motif	TCTTAC	part of a light responsive element
IpPAL5	TCT-motif	TCTTAC	part of a light responsive element
IpPAL5	TCT-motif	TCTTAC	part of a light responsive element
IpPAL5	TCT-motif	TCTTAC	part of a light responsive element
IpPAL5	TCT-motif	TCTTAC	part of a light responsive element
IpPAL4	AE-box	AGAAACAA	part of a module for light response
IpPAL3	Box III	atCATTTTCACt	protein binding site
IpPAL1	HD-Zip 3	GTAAT(G/C)ATTAC	protein binding site
IpPAL1	HD-Zip 3	GTAAT(G/C)ATTAC	protein binding site
IpPAL4	WUN-motif	AAATTTCCT	wound-responsive element

Table S5. Ka, Ks, and Ka/Ks ratios of duplicated IpPAL gene pairs in *I. polycarpa*.

Gene 1	Gene 2	Ka	Ks	Ka/Ks	Duplication type
IpPAL1	*IpPAL2*	0.020006316	0.189453147	0.105600335	Segmental duplication
IpPAL1	*IpPAL3*	0.098608411	1.854076464	0.053184652	Segmental duplication
IpPAL1	*IpPAL4*	0.105293159	2.395168513	0.043960648	Segmental duplication
IpPAL2	*IpPAL3*	0.097767958	1.688953420	0.057886711	Segmental duplication
IpPAL2	*IpPAL4*	0.104749500	2.652646361	0.039488679	Segmental duplication
IpPAL3	*IpPAL4*	0.022041009	0.279983774	0.078722451	Segmental duplication

Ka/Ks < 1 is consistent with purifying selection.

Table S6 The expression of IpPALs under the infection of *Botryosphaeria dothidea*.

Gene Name	CCK-1	CCK-2	CCK-3	C24-1	C24-2	C24-3	C96-1	C96-2	C96-3	ZCK-1	ZCK-2	ZCK-3	Z24-1	Z24-2	Z24-3	Z96-1	Z96-2	Z96-3
IpPAL1	76.61	46.31	132.36	92.69	79.82	137.70	166.83	160.27	155.58	87.41	113.00	79.26	109.90	164.24	110.69	126.87	136.55	97.53
IpPAL2	100.69	86.01	157.32	302.98	320.81	439.73	536.22	621.94	552.47	141.42	177.40	133.55	412.88	518.63	378.57	454.14	456.19	416.16
IpPAL3	35.03	17.65	52.87	66.86	101.12	111.41	130.13	168.36	113.95	40.83	36.54	53.21	115.72	166.62	111.42	77.87	95.65	78.29
IpPAL4	34.39	36.36	43.34	68.96	158.60	158.08	138.51	128.76	89.62	39.69	53.07	40.40	124.29	186.12	129.27	114.28	135.84	100.87
IpPAL5	42.91	47.96	44.81	34.60	89.57	84.18	64.99	57.52	63.27	38.76	54.40	30.09	68.72	165.09	87.71	137.70	161.13	124.18

Table S7. Differential expression of IpPAL genes in resistant and susceptible *Idesia polycarpa* provenances following *Botryosphaeria dothidea* inoculation.

Gene	CCK_vs_C24	CCK_vs_C96	ZCK_vs_Z24	ZCK_vs_Z96	CCK_vs_ZCK	C24_vs_Z24	C96_vs_Z96
IpPAL1	--	1.264	0.643	--	--	--	-0.587
IpPAL2	1.849	2.651	1.715	1.763	--	--	--
IpPAL3	1.625	2.316	1.778	1.160	--	--	-0.883
IpPAL4	1.952	1.979	1.906	1.608	--	--	--
IpPAL5	--	0.777	1.573	1.988	--	--	1.020
